# Multilayered Regulation of Fungal Phosphate Metabolism: From Molecular Mechanisms to Ecological Roles in the Global Phosphorus Cycle

**DOI:** 10.3390/life15111676

**Published:** 2025-10-28

**Authors:** Yanan Tan, Yanda Ning, Siyi Wang, Faqin Li, Xuewei Cao, Qin Wang, Ang Ren

**Affiliations:** Sanya Institute of Nanjing Agricultural University, Key Laboratory of Agricultural Environmental Microbiology, Ministry of Agriculture, Department of Microbiology, College of Life Sciences, Nanjing Agricultural University, Nanjing 210095, China

**Keywords:** fungi, phosphate, phosphorus cycle, PHO signaling pathway

## Abstract

Phosphates are essential nutrients for living organisms, and they are involved in various biological processes, including lipid metabolism, energy synthesis, and signal regulation. Recent studies have elucidated the fundamental components and transport proteins of phosphate signaling pathways, thereby providing a more profound understanding of phosphate metabolism in fungi. In this review, we concentrate on synthesizing the recent findings concerning phosphate metabolism in fungi over the past five years. These findings include the role of phosphates in the global phosphorus cycle, their effect on fungal growth and development, the variations in PHO signaling pathways among different species, and their pivotal role in symbiosis with plants. A mounting body of research substantiates the notion that phosphates play a pivotal role in regulating fungal life activities through a multifaceted mechanism. This regulatory function encompasses the promotion of growth and development, adaptation to environmental variations among different fungal species, and the evolution of distinct regulatory factors and transport proteins. Consequently, this fosters fungal diversity.

## 1. Introduction

As a fundamental element of life processes, phosphate is not only a core molecule in energy metabolism and signal transduction but also a key limiting factor for cell proliferation and environmental adaptation. Recent studies have revealed that fluctuations in phosphate levels are closely associated with metabolic reprogramming, membrane lipid synthesis, and stress responses, exhibiting distinct regulatory patterns across different kingdoms of life. In mammals, phosphate signaling pathways involved in bone formation and tumorigenesis have been elucidated [1,2], while in plants, phosphate starvation signals have been linked to root architecture remodeling and reproductive transitions [3,4]. Compared with these well-studied systems, research on phosphate signaling and metabolic regulation in fungi remains relatively limited, particularly regarding transmembrane sensing, energy integration, and ecological interactions. These questions have become critical frontiers in current fungal physiology and environmental microbiology research.

Recent studies have shown that phosphates serve as structural and metabolic molecules in fungi. Their availability also plays a crucial role in pathogenicity, environmental adaptation, cellular structure, and ecological interaction capabilities. In phosphorus-deficient environment, *Candida albicans* showed reduced pathogenicity, weakened tissue invasiveness and poor adhesion [5]. In addition, *Candida albicans* in phosphorus-deficient environment were significantly more sensitive to metal ions, such as iron and copper, and were more susceptible to inhibition by metal toxicity, with retarded cell growth and abnormal membrane structure [6]. Lipid metabolism is also affected by phosphorus levels, and phosphorus deficiency can alleviate physiological stress by regulating the glycerophosphorylcholine (GPC) synthesis pathway and maintaining membrane lipid synthesis [7]. In studies of *Cryptococcus neoformans*, it was found that under conditions of phosphorus deficiency, phosphorus supply affects the formation of fungal pod membranes, and the drug caspofungi, which is used against this fungus, exhibits a more pronounced sensitivity, with a weakening of the associated stress response and a decrease in the cellular ability to resist host phagocytosis [8].

Phosphate is involved in the synthesis of a wide range of metabolites, especially organic acids that are closely related to environmental adaptation, and in fungi of the genus Penicillium, phosphorus deficiency induces oxalic acid and citric acid, which enhance the efficiency of utilization of external phosphorus sources, but also has the potential to trigger extracellular acid stress [9]. *Rhizophagus irregularis*, a symbiotic fungus with plants, has increased dependence on host plants, better symbiosis, and limited expansion capacity under low-phosphorus environmental conditions, and at the symbiotic interface, the ability to take up and transport phosphorus is directly related to the survival of the symbiotic fungus [10].

Fungi, especially mycorrhizal fungi, are key drivers of global phosphorus cycling, participating in phosphorus transformation, redistribution and nutrient regulation in ecosystems. In terrestrial ecosystems, over 80% of vascular plant species form symbiotic associations with mycorrhizal fungi, particularly with arbuscular mycorrhizal fungi [11]. Fungi facilitate the mineralization of both organic and inorganic phosphorus in the environment through the release of organic acids and acid phosphatases. This process results in the release of phosphorus that is low in availability in the soil and the acceleration of the rate of phosphorus acquisition by plants. Consequently, fungi contribute to the biochemical geocycling of phosphorus in the land surface [12,13,14]. Ectomycorrhizal fungi play a pivotal role in the transport and storage of phosphorus in temperate and boreal regions. These organisms expand the availability of plant phosphorus sources within forest ecosystems by establishing a network of highly differentiated mycelia and by facilitating the mobilization of deep and heterogeneous phosphorus pools [15]. Furthermore, fungi have been shown to accelerate phosphorus mineralization and recycling through the processes of biodegradation and soil microbial collaboration [16]. Concurrently, mycorrhizal types, functional diversity, and soil conditions collectively influence the regional and overall distribution patterns of phosphorus and the pathways through which nutrients are exported [17,18,19]. Fungi function as mediators of plant phosphorus uptake and play a pivotal role in regulating and connecting elements of the global phosphorus cycling network.

**Figure 1:**
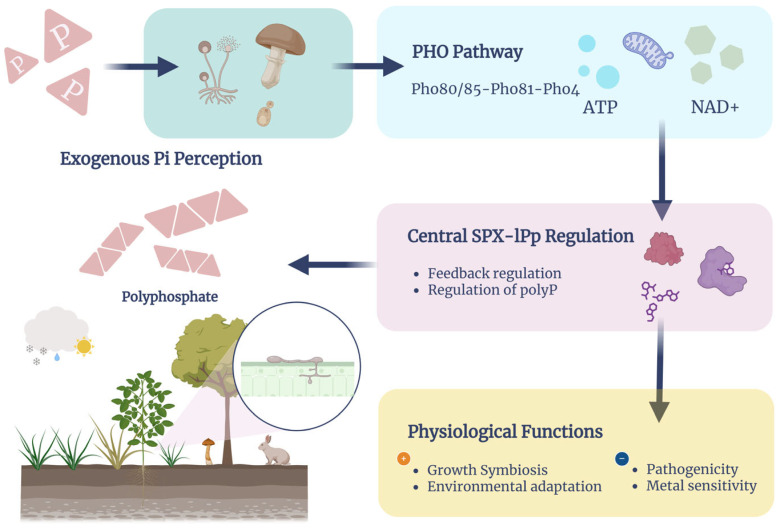
Fungal phosphate metabolism and adaptation [1,3,5,13,15]. The system has a positive effect (+) by promoting growth, symbiosis, and environmental adaptation, and a negative effect (−) by suppressing pathogenicity and affecting metal sensitivity. (Created in BioRender. YD, Ning. (2025) https://app.biorender.com/illustrations/canvas-beta/68aeb489bcafd753d7606460, 12 September 2025).

Fungi regulate intracellular phosphorus homeostasis through the highly conserved PHO signaling pathway by sensing exogenous phosphorus levels. Taking *Saccharomyces cerevisiae* as an example, the cell cycle protein-dependent kinase Pho85 and Pho80 together form the CDK complex, and the inhibitor of the CDK complex Pho81 is inactivated when there is sufficient phosphorus, and the CDK complex acts on the transcription factor Pho4, which is phosphorylated and localised in the cytoplasm, When Pho4 is phosphorylated and cytoplasmically localized, it cannot act on downstream genes and related proteins; when phosphorus is deficient, Pho81 binds to the CDK complex, Pho4 is released, dephosphorylated and localised in the nucleus, activating phosphorus transporter and phosphatase genes such as Pho84 and Pho5 [20] (Figure 1).

In recent years, research on fungal phosphate metabolism has continued to deepen, with important advances in polyphosphate regulation, cross-boundary signalling integration and plant interactions mechanisms. In the tufted mycorrhizal fungus, such as *Rhizophagus irregularis*, it was found that the SPX structural domain transporter protein RiPT7 regulates the transmembrane transport of polyphosphate at the symbiotic interface, which affects the phosphorus storage within the hyphae and the efficiency of phosphorus supply to plants [10]. Inositol pyrophosphate metabolites, especially 1,5-IP8, bind to the SPX structural domain and transmit cellular phosphorus sufficiency signals by regulating Pho81 activity, and studies have also found a relationship between the phosphate sensing system and NAD+ metabolism [21]. In addition, some researchers used high-throughput macro-genome sequencing to explore the phosphorus metabolism gene network of mycorrhizal fungi and soil microorganisms around the mycelium, and for the first time resolved the synergistic regulatory process of key genes, such as PhoP, PhoD, gcd, and pst, in macroscopic, which revealed the functional correlation of phosphatase activity stimulated by *R. irregularis* [13]. Combined transcriptome and metabolome analyses in the fungus *Trametes gibbosa* revealed that the strain initiated the differential expression of multiplex metabolic pathways under the stimulation of different phosphorus sources, and resolved the mechanism of phospholysis [22]. Through the combination of structural biology and gene editing, the mechanism of interaction between small molecules and transporter proteins can be precisely analysed [7].

Research into fungal phosphorus metabolism and its role in ecosystem phosphorus cycling is advancing rapidly, with most studies centred on model yeasts and mycorrhizal fungi. The cross-regulatory mechanisms linking fungal phosphate signalling systems—including the PHO pathway, SPX domains, and inositol pyrophosphate—to cellular energy metabolism and epigenetic regulation remain poorly understood. The role of polyphosphate in the regulation of intracellular homeostasis and in host–fungal interactions has not yet been systematically elucidated. Existing literature predominantly addresses molecular mechanisms and species-level interactions, with insufficient integration from molecular mechanisms to ecological functions.

This review systematically outlines the molecular regulatory mechanisms and ecological functions of fungal phosphate metabolism, particularly its roles in environmental adaptation, ecological interactions, and the global phosphorus cycle. By synthesising the recent advances in fungal phosphate sensing and transcriptional regulation over the past five years, it elucidates the relationship between the PHO signalling pathway, the relationship between the SPX domain and inositol pyrophosphate, and molecular variations across fungal species. The present study provides further elucidation on the role of polyphosphate metabolism and transmembrane transport in the maintenance of cellular homeostasis and energy metabolism. The review explores the functional significance of fungal phosphate metabolism in symbiosis, pathogenicity, and ecosystem phosphorus cycling, while assessing its potential applications in agriculture and biotechnology. The objective of this review is to further enhance the research framework on the PHO signalling pathway and phosphorus cycling, thereby providing a novel theoretical foundation for developing innovative approaches to fungal phosphate metabolism in ecological and biotechnological applications, as well as for the sustainable utilisation of phosphorus.

## 2. Phosphate-Sensing and Transcriptional Regulation Mechanism in Fungi

### 2.1. Pho Signalling Pathway and Transmembrane Sensing

Inorganic phosphate (Pi) sensing and transport in fungi rely on the classic PHO pathway, a system that not only regulates transcription but also deeply integrates Pi transport, storage and metabolism. Recently, it was found that the “perfect adaptation” to low phosphorus in *S. cerevisiae* does not rely on conventional negative feedback, but is determined by the transport limit of the transporter proteins, which enables homeostatic control of Pi levels in the cell [23]. Pho89 is a Na^+^-dependent high-affinity transporter adapted to the needs of Pi uptake in alkaline environments. Pho90, although of lower affinity, is not limited to transporter function but is also closely related to intracellular polyphosphate storage and regulation. It is also closely related to intracellular polyphosphate storage and regulation, and is regulated by Spl2, which is induced by the PHO regulatory system and then inhibits phosphorus transport by binding to Pho90 [24]; Pho81 interacts with Pho90 through its SPX structural domain to localise Pho90 in eukaryotic vesicles under phosphorus-deficient conditions, and maintains its stability through the SPX structural domain [25].

In addition to transporter proteins, Pho84 expression is also interactively regulated with other environmental factors, such as metal ion stress, and aluminium stress significantly inhibits PHO84 expression and induces phosphorus starvation, deepening the understanding of the environmental response mechanism of the PHO pathway [26]. Evolutionarily, Pho7 replaces Pho4 in fission yeast to dominate PHO promoter regulation, and the systematic structure–function divergence from *S. cerevisiae* is obvious. Pho7 directly binds to the target promoter, and responds synergistically with multiple regulatory factors triggered by phosphorus starvation [27,28]. Shp2, a putative polyamine transporter protein in fungi, promotes phosphate efflux and is independent of Xpr1, a known phosphate efflux protein, which helps maintain phosphate homeostasis in high-phosphorus environments and enhances tolerance to high-phosphorus stress, revealing novel phosphorus regulatory pathways in addition to the traditional PHO pathway [29]. Abnormalities in the PHO pathway also affect energy metabolism and pathogenicity, making it a potential target for novel antifungal strategies. Abnormalities in the PHO pathway also affect energy metabolism and pathogenicity, making it a potential target for novel antifungal strategies, and its role in symbiotic systems is supported by the regulation of Pi uptake by a similar Pho4 homologue in AM.

The PHO pathway is a highly integrated Pi-sensing and uptake system through a series of transmembrane transport proteins (e.g., Pho84, Pho89, Pho90) and their regulators (e.g., PHM6, PHM7, Pho81, Spl2), which dynamically responds to changes in exogenous Pi and takes into account the regulation of endogenous polyP storage. The structural and functional studies have gradually revealed the logic of the PHO pathway’s “sensing-transporting-signalling” trinity, which provides a key perspective for the analysis of eukaryotic phosphorus metabolism [30].

Recent studies have revealed the diversity and complexity of the PHO signalling pathway in different fungi. This pathway plays a central role in phosphorus sensing and transcriptional regulation, and is also closely linked to energy metabolism, cellular stress responses and ecological adaptation. Additionally, variations specific to different species exist within this pathway, as summarised briefly in existing literature (Table 1). While the core regulatory mechanisms in model yeasts are relatively well understood, many unanswered questions remain in most non-model fungi. These include the molecular basis of transmembrane sensing, variations in Pho4 phosphorylation regulation and cross-regulatory mechanisms between the PHO signalling pathway and other metabolic networks. Future research must urgently integrate omics analyses, structural biology, and gene editing technologies to elucidate the dynamic regulatory patterns of fungal PHO signalling pathways in complex environments and their ecological significance.

**Table 1:** Comparative overview of the PHO signaling pathways across representative fungal species.

Species	Core Transcription Factor	Upstream Regulatory Factors	Molecular Mechanism
*Saccharomyces cerevisiae*	Pho4 (bHLH transcription factor)	Pho80–Pho85 complex; Pho81 (CDK inhibitor protein); Pho84 (Pi transporter)	Under low-phosphorus conditions, Pho81 inhibits the Pho80–Pho85 kinase complex. Following dephosphorylation, Pho4 enters the nucleus, where it binds to Pho2 to activate PHO gene transcription [31,32,33].
*Schizosaccharomyces pombe*	Pho7 (Zn^2+^-Cys_6_-type transcription factor)	Cdkp1–Pho80–Pho85-like complex; Asp1 (IP8 synthase); SPX protein	Pho7 directly recognizes low-phosphorus response gene promoters; IP8 regulates transcription responses by modulating SPX proteins, employing a non-homologous mechanism distinct from that in *S. cerevisiae* [27,34,35].
*Candida albicans*	Pho4 (regulatory factor)	Pho80–Pho85–Pho81 complex; Pho84	Pho4 nuclear localization is regulated by Pho85 phosphorylation; under low-phosphorus induction, Pho4 activates phosphatase genes and enhances cell wall and oxidative stress tolerance [35].
*Cryptococcus neoformans*	Pho4	Pho85–Pho80–Pho81 homologs; SPX–IP7 regulatory axis	Pho4 regulates phosphorus uptake and virulence-related genes; IP7 interacts with the SPX domain to stabilize the Pho pathway, influencing fungal survival within the host.
*Aspergillus nidulans*	PhoP (Zn^2+^-Cys_6_ transcription factor)	PhoR–PhoS two-component system; SPX domain protein; VTC complex	PhoP is regulated by PhoR–PhoS signaling, controlling phosphate uptake and polyphosphate metabolism; it cross-regulates with secondary metabolism and developmental pathways.

### 2.2. Inositol Pyrophosphate and the Mechanism of SPX Structural Domain Regulation

In recent years, inositol pyrophosphates (IPPs) such as IP7 and 1,5-IP8 have been identified as core signalling molecules regulating the fungal Pi response, which regulate the function of proteins such as Pho81 and Vtc4 by binding to the SPX structural domains, and act as a second sensor for the initiation of the PHO signalling pathway [36]. Decreasing levels of 1,5-IP8 specifically in response to phosphorus starvation at the transcriptional level, i.e., the PHO signalling A decrease in 1,5-IP8 levels specifically triggers the phosphate starvation response at the transcriptional level, i.e., the PHO signalling pathway, and a decrease in 1,5-IP8 levels leads to activation of Pho81 and deregulation of Pho4 inhibition, which rapidly triggers expression of phosphorus-responsive genes. Excessive or absent levels of 1,5-IP_8_ cause aberrant expression of PHO target genes, e.g., pho1.

The metabolic enzymes of the inositol pyrophosphate metabolic pathway, Aps1, Asp1, and Kcs1, are important regulators of the Pi response, and these enzymes regulate IP8 homeostasis, and their dysregulation can lead to abnormal activation of the PHO pathway and toxicity accumulation [37,38]. Asp1, a bifunctional inositol pyrophosphorylase specific to fission yeast, possesses the ability to phosphorylate 5-IP_7_ to 1,5-IP_8_, and dephosphorylates 1,5-IP_8_ [39]. Asp1 is a Nudix-family inositol pyrophosphatase that efficiently degrades a wide range of IPP molecules, particularly 1-IP_7_ and 1,5-IP_8_, and has a central regulatory role in phosphate signalling. Deletion of Aps1 causes accumulation of IP_8_ levels and leads to non-physiological high expression of PHO genes and cytotoxicity and plays a critical role in maintaining IP_8_ homeostasis and buffering signal strength. Asp1, Siw14, and Aps1, three inositol pyrophosphate hydrolases, are functionally redundant in fission yeast and work together to maintain a dynamic balance of IPP levels. Tandem inactivation of the three enzymes leads to IP8 accumulation, cell cycle disruption, phosphorus metabolism imbalance, and stress sensitivity, further emphasising the importance of the dephosphorylation mechanism of IPPs for the regulation of cellular homeostasis and PHO pathways [40].

Genetic repressor screening revealed that glycerophosphocholine transporter protein Tgp1, IP6 kinase Kcs1 and phospholipase C Plc1 are key factors regulating the toxic accumulation of fission yeast inositol pyrophosphate. Studies have shown that Tgp1 affects the transport of cell membrane-related metabolites and indirectly regulates the homeostasis of IPPs; Kcs1, as an IP6 kinase, catalyzes the synthesis of IP7 and IP8, which is the core of the metabolism of IPPs and signaling; and Plc1 regulates the dynamic balance of intracellular signaling phospholipids, and the synergistic action of the three factors effectively mitigates cytotoxicity triggered by the aberrant accumulation of IPPs, which represents the complex regulation of the metabolic network of IPPs. These three synergistic effects effectively alleviate the cytotoxicity caused by the abnormal accumulation of IPPs, reflecting the complex regulatory mechanism of multiple factors in the IPPs metabolic network and providing a new molecular perspective for the regulation of the PHO pathway [38]. In *S. cerevisiae*, Kcs1 acts as a major IP_6_ kinase, catalysing the generation of IP_7_, which is an important upstream enzyme for Pho81 signalling of inositol pyrophosphates (IPPs), while Vip1 acts as a bifunctional kinase-phosphatase, regulating the synthesis and degradation of IPPs and maintaining 5-IP levels. The activity of Kcs1 is regulated by phosphorylation of the energy-sensing kinase Snf1, which forms a bridge coupling energy state and phosphate signalling and coordinates cellular metabolism and phosphorus response [41]. Absence of Kcs1 leads to aberrant activation of the PHO pathway, affecting the expression of target genes such as PHO5.

**Figure 2:**
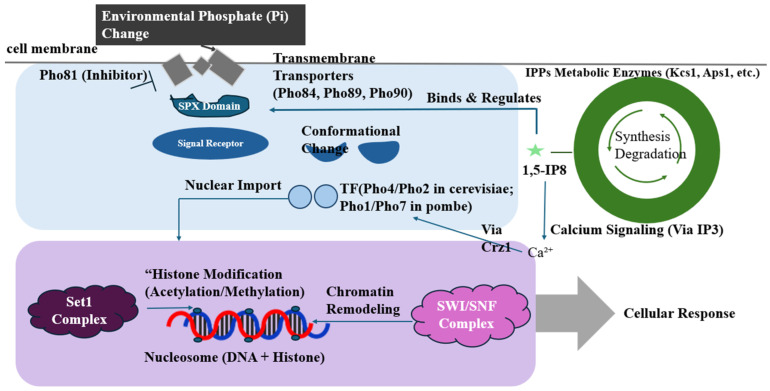
Phosphate sensing and transcriptional regulation in fungi [28,42,44,48,52].

As a signal integration module on several key proteins (e.g., Pho81, Vtc4, Spl2), the SPX domain can bind IPPs with high affinity to achieve precise sensing and regulation of phosphorus signals, and Kcs1 and Vip1 jointly regulate the function of the SPX domain of Pho81, which can regulate the activation and inhibition of the PHO signalling pathway and safeguard the adaptive capacity of the cell to phosphorus starvation and environmental changes. This mechanism reveals the complex regulatory network of IPPs metabolism and its coupling with energy metabolism, which provides a key molecular basis for the in-depth understanding of fungal phosphorus metabolism and its physiological functions [42]. The SPX domain not only regulates Pho81, but also assists the synthesis of polyP in the vesicle membrane by the VTC complex, which is a key mechanism for Pi storage [43]. IPPs also interact with calcium signalling, and the intracellular calcium released by IP3 induces the release of calcium via the calmodulin. IP3 induces the release of intracellular calcium, which can affect the expression of Pho89 through the calmodulin Crz1, constituting the “IP-calcium-Pi” ternary regulatory system [44]. In fission yeast, the splicing-polyadenylation factor Cft1, together with the SPX structural domain proteins, is involved in the regulation of the toxic accumulation of inositol pyrophosphates, which prevents cytotoxicity by restricting the excess of IPPs, demonstrating the negative feedback mechanism between the PHO pathway and the metabolism of IPPs [45] (Figure 2).

It has been demonstrated in previous studies that the SPX domain and inositol pyrophosphate metabolism exhibit a conserved signal integration function within the phosphorus sensing system. However, further research is required to elucidate the functional differences in fungi, ligand selectivity, and transmembrane cooperative mechanisms. It is recommended that future research concentrate on the structural diversity of SPX proteins across different fungi and their coupled regulation with cellular energy status. This will facilitate the revelation of deeper connections between phosphorus signalling and metabolic homeostasis.

### 2.3. Epigenetic Regulation of the PHO Pathway

The epigenetic regulatory mechanisms of the PHO pathway have gained in-depth attention in recent years, involving a variety of mechanisms such as chromatin remodelling, antisense RNA, transcription termination, and NAD^+^ metabolic coupling.

In fission yeast, inositol pyrophosphate signalling achieves epigenetic negative regulation of the PHO pathway through the mediation of chromatin remodelling factors. High IP8 levels assist in the construction of a repressive chromatin state in the PHO promoter region via Snf22/Sol1 in the SWI/SNF chromatin remodelling complex, thereby preventing the binding and activation of the key transcription factor Pho7 to the target promoter, and delaying the induction of the Pho1 response double mutants of Asp1 with chromatin remodelling factors, such as Snf22, exhibit significant synthetic lethality, suggesting that they indirectly affect the expression of phosphate-responsive genes by regulating chromatin conformation [46]. These findings reveal the central regulatory function of chromatin remodelling factors in the phosphate signalling pathway and provide experimental evidence for the coupling mechanism between phosphorus signalling and epigenetic regulation [47].

In *Saccharomyces cerevisiae*, even in the absence of the SWI/SNF complex, which is thought to be essential for chromatin remodelling, its PHO8 and PHO84 promoters can still achieve nucleosome remodelling and activate transcription, suggesting a high degree of plasticity and redundancy in the chromatin remodelling mechanism of the PHO pathway. It is suggested that alternative chromatin remodelling factors (e.g., INO80 or ISW1) may mediate the formation of the promoter open state in the context of SWI/SNF deletion and assist in the binding of transcription factors such as Pho4. This result challenges the conventional view of SWI/SNF as a factor essential for chromatin remodelling of the PHO pathway and suggests the existence of more flexible remodelling pathways for eukaryotic gene expression regulation [48]. Fission yeast exhibits large-scale transcriptome remodelling under phosphorus starvation conditions, where Pho7, as a functional analogue of Pho4, dominates the activation of key phosphorus-responsive genes and acts synergistically with chromatin regulators, such as the Set1 complex, to determine promoter activation efficiency. These epigenetic factors not only regulate the binding affinity of Pho7 to target promoters but also play an important role in phosphorus starvation-induced lifespan extension, suggesting a coupling between the PHO pathway and cellular metabolic fate. It was shown that Pho7-driven response mechanisms prompted glycolytic and oxidative metabolic pathway remodelling and prolonged the chronological lifespan of fission yeast, further highlighting the association between phosphorus-sensing signalling and aging mechanisms [49].

The effects of coding and non-coding transcripts on the expression of phosphate-responsive genes in *Saccharomyces cerevisiae* were analysed by the RNA sequencing system. The promoter regions of several key genes of the PHO pathway (e.g., PHO84, PHO5) have obvious bi-directional transcriptional characteristics, producing transient and unstable non-coding transcripts (antisense RNA); these non-coding transcripts will competitively occupy the promoter regions during the phosphorus starvation response. These non-coding transcripts compete to occupy the promoter region during phosphorus starvation and interfere with the binding of Pho4 and other transcriptional activators through transcriptional interference, leading to a decrease in the expression level of target genes. This regulatory mechanism is highly site-specific and dependent on the activity of the intranuclear RNA degradation system, e.g., loss of function of the nuclear exosome component Rrp6 leads to the stable accumulation of antisense RNA and reinforces the repression of PHO target gene expression. This finding complements previous studies on the mechanisms by which non-coding RNAs mediate chromatin regulation. For example, Camblong et al. found that accumulated antisense RNA recruits the histone deacetylase complex to the PHO84 promoter region, causing chromatin compression and repressing sense strand transcription [50]. In addition, the Nrd1-Nab3-Sen1 complex acts as an early termination factor, regulating the termination and degradation of noncoding transcription and limiting the diffusion of antisense RNA, thereby protecting the normal expression of PHO pathway genes [51].

The production and stabilisation of such non-coding RNAs and their effects on chromatin structure build a multilevel network of PHO pathway regulation. As research continues to advance, it has been discovered that the PHO signalling pathway in fungi is not only governed by classical transcriptional regulatory elements but is also intricately intertwined with epigenetic modifications. The dynamic alterations in histone acetylation and deacetylation during phosphorus starvation responses reveal a multi-tiered regulatory mechanism for the transcriptional activation of Pho4 and its target genes.

Despite the evidence provided by earlier studies that deacetylases such as Hst1 and Rpd3 influence PHO pathway activity by regulating NAD+ metabolic pathways, the question of the conservation and variability of their mechanisms across different fungi remains unresolved. Moreover, the coupling relationship between epigenetic modifications and inositol pyrophosphate (IPP) and polyphosphate (polyP) metabolism has yet to be systematically elucidated. Future research should integrate chromatinomics, metabolomics, and single-cell technologies to decipher the spatiotemporal characteristics and network dynamics of epigenetic regulation during fungal phosphorus response.

## 3. Phosphate Metabolism and the Synergistic Network of Homeostasis in the Body

### 3.1. Synthesis and Regulatory Mechanisms of Polyphosphate

Polyphosphate is a linear polymer widely found in living organisms, consisting of tens to hundreds of orthophosphate groups linked by high-energy phosphate ester bonds [53]. In fungi, polyP is primarily stored in vacuoles to provide cells with available Pi and to respond to phosphorus starvation and stress. Recent studies have revealed that the coupling mechanisms between polyphosphate metabolism and cellular metabolic networks are highly complex, involving multiple enzymes, membrane transporters, and signaling pathways, among other biological processes. This further confirms the biological significance of polyP within cells.

In fungi, polyP is synthesized by the VTC complex, which is dependent on the vacuolar membrane. The VTC complex consists of five subunits, VTC1-5, with VTC4 being the key subunit containing polyphosphatease activity; its absence leads to impaired polyP biosynthesis [54,55]. The VTC complex catalyzes polyP synthesis in the cytoplasm and transports polyP into the vacuole through a channel formed by the complex. Cryo-EM analysis of the VTC complex revealed that VTC4, VTC3, and VTC1 assemble in a 3:1:1 ratio in yeast and form a novel polyP-selective channel through 15 transmembrane helices [54]. When the VTC complex is in an activated state, the substrate ATP is converted into polyP by the catalytic domain at the channel entrance and then transported into the vacuole via immediate transmembrane transfer, completing the synthesis and transport process [43].

Inositol pyrophosphate precisely regulates the VTC complex. Subunits VTC2 and VTC3 contain SPX domains that sense the presence of inositol pyrophosphate and bind to it to regulate the activity of the complex [25]. Recent studies have revealed that the SPX domain of the VTC2 subunit can inhibit the polyphosphatase activity of VTC4 through homologous SPX-SPX interactions. When inositol pyrophosphate binds to VTC2, it alleviates this inhibitory effect, enabling the VTC complex to enter an activated state and promote polyP biosynthesis. PolyP stored in vacuoles is degraded by the participation of multiple phosphatases, releasing available inorganic phosphorus for cellular metabolic activities. For example, in yeast cells, the exopeptidase PPX1 sequentially cleaves inorganic phosphorus from the polyP terminals, while the endopeptidase PPN1 and the two-subunit enzyme Ddp1 can cleave high-molecular-weight polyP into shorter polymers. New research has found that the polyphosphate phosphatase Ppn2 is induced under alkaline conditions to degrade polyP and neutralize high intracellular pH [56,57,58]. The coordinated action of polyP synthases and degradative enzymes achieves dynamic balance by synthesizing and storing polyP under phosphorus-sufficient conditions and accelerating degradation under phosphorus-deficient conditions. Additionally, the VTC complex is closely associated with the Pho signaling pathway. When phosphorus is deficient in cells, the Pho signaling pathway is activated, and the transcription factor Pho4 regulates the expression of downstream-related genes, including key genes in the VTC complex. The biosynthesis and degradation of polyP are subject to multifaceted synergistic regulation, playing an irreplaceable role in phosphorus supply during biological activities.

### 3.2. Intracellular Transport of Phosphate

In addition to transporters localized on the cell membrane, transporters are also present on the vacuole membrane to facilitate the utilization of polyP stored in the vacuole. PHO91 is a typical intracellular transporter localized on the vacuole membrane. The yeast low-affinity phosphate transporter PHO91 contains an SPX domain at its N-terminus, which regulates transport function [59]. The SPX domain of PHO90 interacts with the regulatory protein Spl2 to limit the rate of phosphate uptake and inhibit phosphate efflux [20,36]. Cryo-EM structures of PHO90 reveal that in the presence of phosphate, the protein adopts an outward-open conformation, while in the absence of phosphate, it adopts a dual-unit conformation with one subunit inside and one outside, providing the structural basis for transmembrane transport [60]. PHO91 is localized on the vacuolar membrane and is responsible for releasing phosphate from the vacuole into the cytoplasm, thereby maintaining intracellular phosphate homeostasis. In PHO91 mutant cells, intracellular polyP levels significantly increase, confirming the transport function of PHO91 [59]. Knocking out the PHO91 gene reduces cellular tolerance to cadmium while enhancing tolerance to manganese and oxidative stress, indicating that PHO91 plays a crucial role in maintaining intracellular ion balance and redox homeostasis [61].

The transporter protein XPR1 found in higher organisms possesses an SPX domain and achieves phosphate efflux by sensing intracellular PP-InsP [62]. A similar phosphate efflux-active Xpr1 has been identified in fission yeast, whose function is co-regulated by the phosphate uptake inhibitor protein Pqr1 and the VTC complex [20]. Shp2 is an additional export pathway complementary to Xpr1, particularly assisting in the removal of excess phosphate in high-phosphate environments to maintain cellular phosphate homeostasis. The synergistic action of these two pathways ensures that cells can flexibly adapt to changes in phosphate levels in the environment, achieving metabolic balance and homeostasis regulation [29]. PHM6 and PHM7 are recently discovered membrane proteins encoding unknown functions that promote phosphate uptake and polyP accumulation under phosphate-sufficient conditions [63]. When the PHO signaling pathway is continuously activated, cells accumulate large amounts of polyP. Deleting any one of the low-affinity transporters PHO87, PHO90, or PHO91 further increases polyP levels, indicating that these pathways normally limit excessive polyP accumulation. This suggests that a negative feedback loop exists between cellular phosphate uptake and intracellular phosphate transport to prevent phosphate supersaturation [59]. Comprehensive research and new findings indicate that fungi achieve intracellular phosphorus balance through the PHO signaling pathway, the VTC complex, and various transport proteins.

### 3.3. Coupling of Phosphate Homeostasis and Cellular Homeostasis

Phosphate participates in various life activities and thus regulates cellular homeostasis, closely associated with cellular energy metabolism. During phosphate starvation, intracellular ATP and inositol pyrophosphate levels significantly decrease. Wild-type cells with sufficient polyP reserves can alleviate this energy-deficient state. Under normal phosphate conditions, polyP-deficient cells also exhibit starvation characteristics and growth defects, indicating that under phosphate-sufficient conditions, polyP also serves as a phosphate source to provide available phosphate for cells [64], supplying phosphate for ATP synthesis and supporting the biosynthesis of nucleotides and ATP. Within cells, polyP functions as a phosphate storage pool to maintain phosphate homeostasis, acting as a phosphate buffer reservoir to achieve dynamic balance of phosphate within cells [65]. This indicates that polyP serves as a phosphate storage pool for phosphate buffering, providing a phosphate source for cellular metabolism and participating in the regulation of cellular homeostasis.

In addition to influencing cellular energy metabolism by participating in ATP biosynthesis, phosphate deficiency may lead to changes in NAD+/NADP+ levels. Under phosphate-deficient conditions where polyP synthesis is impaired due to VTC4 mutations, cellular NAD+ levels decrease, accompanied by changes in intermediate metabolites of the tryptophan metabolic pathway [64]. The availability of phosphorus affects signaling pathways such as TOR and PKA, thereby influencing the expression and activity of NAD+ synthase and synergistically regulating NAD+ levels [66,67].

Polyphosphate also participates in regulating metal ion homeostasis. Multivalent cations such as Zn^2+^, Ca^2+^, and Mg^2+^ are chelated by polyP to form complexes [68]. A decrease in polyP levels affects Mg^2+^ uptake [55], leading to PHO80 inactivation. Cells accumulate large amounts of phosphorus and polyP, accompanied by abnormal accumulation of zinc, magnesium, and other metal ions within the cell, resulting in increased sensitivity to metal stress. Although PHO81 deficiency leads to a decrease in phosphorus and polyP, it does not affect metal ion chelation or stress sensitivity [52]. PolyP metabolic dysfunction indirectly affects intracellular metal homeostasis and redox balance [55].

Phosphate homeostasis is also associated with lipid metabolism. PolyP affects cellular lipid metabolism and membrane structural integrity. In *Candida albicans*, a mutant lacking the kinase VIP1, which synthesizes 1-IP7 and participates in activating the PHO signaling pathway, exhibits reduced PHO signaling, leading to excessive energy metabolic stress within the cell. The cell requires more phosphate to participate in metabolism, resulting in abnormal lipid droplet accumulation and membrane damage under phosphate-deficient conditions. Supplementing exogenous phosphate or enhancing phosphate transport can alleviate these phenomena, but metabolic activity does not recover [69]. Further studies revealed that the transcription factor PHO4 is mislocalized and degraded in vip mutant cells, impairing the PHO signaling pathway.

**Figure 3:**
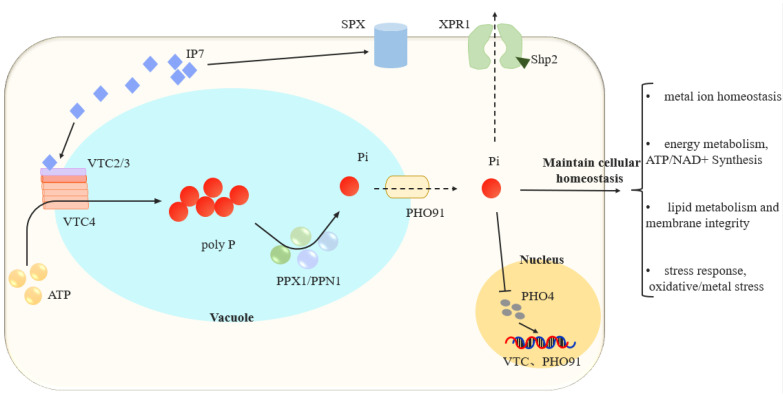
Synergistic network of phosphate metabolism and cellular homeostasis [48,50,51,56,59]. The solid arrow (→) indicates conversion or activation; the dashed arrow (- - →) indicates transport; the T-bar arrow (⊥) indicates inhibition. In the vacuole, the VTC complex (VTC2/3, VTC4) synthesizes polyphosphate (poly P) using ATP, while PPX1/PPN1 hydrolyze poly P into inorganic phosphate (Pi). Pi is released to the cytoplasm via PHO91 and exported by XPR1. Cytoplasmic Pi regulates this pathway: high Pi inhibits the nuclear entry of PHO4, whereas low Pi allows PHO4 to enter the nucleus and activate *VTC* and *PHO91*, forming a feedback loop. IP7 and SPX proteins participate in this regulation, which is essential for maintaining metal ion balance, energy metabolism, lipid metabolism, and stress response.

Polyphosphate participates in cellular energy metabolism, influencing mitochondrial function and cell membrane integrity, and is involved in ATP synthesis, NAD+ metabolism, metal ion sensitivity, and lipid metabolism, thereby contributing to cellular metabolic homeostasis and energy balance (Figure 3).

## 4. Fungal Environmental Interactions and Ecological Functions Regulated by the Phosphate Signalling Pathway

### 4.1. Ecological Status of Fungi in the Global Phosphorus Cycle

Phosphorus is an important nutrient element in living organisms and is involved in many life activities, such as the biosynthesis of nucleic acids, ATP, and the biosynthesis of cell membrane constituents.

The global phosphorus cycle consists of mineral weathering to release inorganic phosphorus, organic phosphorus mineralisation and phosphorus precipitation and remobilisation, in which fungi are involved in promoting the phosphorus cycle through a variety of mechanisms. Mineral weathering is the main source of inorganic phosphorus, which exists in soil in insoluble forms, such as apatite minerals bound to ions such as Al^3+^, Fe^3+^, Ca^2+^, etc., and has low bioavailability [70]. Fungi can attach to the mineral surface through mycelium, secrete low molecular weight organic acids such as oxalic acid and citric acid, chelate metal ions to promote the dissolution and release of insoluble mineral phosphorus, reduce the local pH, and synergistically activate the extracellular phosphatase to achieve the efficient mobilisation of phosphorus, and in farmland soil [71], it is found that the fungi such as *Aspergillus niger* and *Penicillium* have highly efficient phosphorus solubilizing ability, which can increase the phosphorus solubilizing capacity of insoluble or non-soluble minerals. ability, which can enhance the release of insoluble or insoluble phosphorus and thus increase the bioavailability of phosphorus. The secreted extracellular polysaccharides, phenolic compounds, and organic acids can promote the nucleation of mineral particles such as carbonates and silicates, and induce the precipitation of phosphate minerals such as calcium phosphate and barium sulphate in the extracellular matrix [72,73]. The wood-decaying fungus *Trametes gibbosa* has been shown to achieve efficient dissolution of both organic and inorganic phosphorus by upregulating alkaline phosphatase (ALP) activity and secreting multiple organic acids [22]. Through biochemical metabolism, it releases insoluble phosphorus, thereby enhancing phosphorus availability to both plants and microbial communities. Moreover, the ecological function of this organism in the decomposition of organic matter promotes the redistribution of phosphorus between decaying wood, soil, and plants. *Phanerochaete chrysosporium*, a prototypical white rot fungus, has been shown to induce acid phosphatase and high-affinity phosphate transport systems under inorganic phosphorus deficiency, thereby facilitating the efficient acquisition of exogenous phosphorus [74]. The subject’s phosphorus metabolism has been demonstrated to be intrinsically linked to fundamental cellular physiological processes, in addition to its profound engagement in ecological functions, including lignin degradation and environmental adaptation. Fungi also convert organic phosphorus from the environment, such as humus and agricultural wastes, into soluble orthophosphate by releasing a variety of hydrolytic enzymes such as acid and alkaline phosphatases.

In different ecosystems, there is a correlation between fungal community structure and phosphatase activity, with dominant strains increasing available phosphorus in the soil by 20% to 40% at high C/P ratios [75]. Fungal cells synthesise long-chain polyP for storage in vesicles under high-phosphate conditions, which, in addition to maintaining intracellular homeostasis, prolongs the use of available phosphorus in the soil and reduces fertiliser use [76]. Fungal–bacterial interactions also improve the efficiency of phosphorus mobilisation, with fungi participating in the phosphorus cycle by recruiting bacteria to work together, the soluble phosphorus produced by the phosphorus solubilizing activities of fungi can be further utilised by phosphorus-solubilizing bacteria, and the bacterial colonies can, in turn, promote fungal reproduction and phosphatase secretion, forming a virtuous cycle of joint participation [70,77]. In addition to the participation of fungi in the phosphorus cycle by themselves and in collaboration with bacteria, mycorrhizal symbiosis is also a key link in the participation of important fungi in the phosphorus cycle.

### 4.2. Promotion of Fungal Phosphorus Response in Plant Symbiotic Interactions

Mycorrhizal symbiosis is also an important link for fungi to participate in the phosphorus cycle, where clumping mycorrhizae produce a mycelial network to provide plants with available phosphorus outside the growth zone, and the phosphatase secreted by fungi can break down insoluble phosphorus in the soil to supply plant growth. The PHR-SPX receptor system in plants initiates the expression of downstream genes when there is insufficient available phosphorus in the soil, and the activation of key transcription factors induces the expression of symbiotic key genes and promotes the formation of symbiotic structures by mycelial infestation [78,79,80], and the phosphate transporter protein RiPT7, which contains an SPX domain, and it is a bi-directional transporter located on the cell membrane, and it not only supplies available phosphorus to the plants but also avoids the accumulation of phosphorus in the fungi, leading to fungal phosphorus poisoning. It was found that RiPT7 mutants showed premature degradation of septa and root vesicles and vesicle reduction in root mycelium during phosphorus deficiency, confirming that phosphorus transfer to the root cell signals the maintenance of mycorrhizal development [10]. The transcription factor RiPHO4, found in the mycorrhizal fungus *Rhizophagus irregularis*, is involved in phosphorus regulation at the symbiotic interface, responding to environmental phosphorus signals and activating target genes through nuclear localisation to maintain mycorrhizal development and phosphorus supply [81]. In tomato, two members of the SPX protein family, SlSPX1 and SlSPX2, were found to regulate mycorrhizal symbiosis and plant growth, and to inhibit the regulation of phosphorus uptake and storage by plant PHR structures [82]. It affects the rate of AM mycelium infestation and the formation of clumplets.

**Figure 4:**
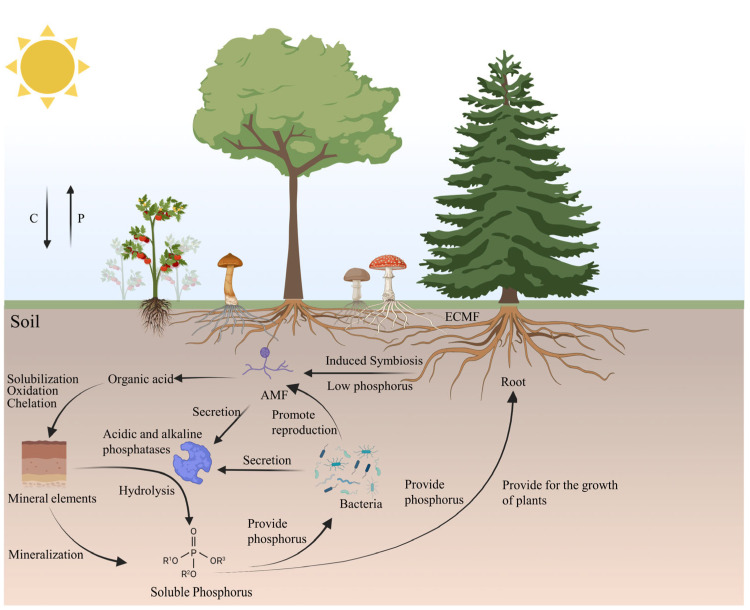
Phosphate signaling in fungal environmental adaptation and symbiosis [72,78] (Created in BioRender. Cao, X. (2025) https://app.biorender.com/illustrations/68af00e13abbdc52dcde1ca9, 12 September 2025).

In addition to the new discovery of molecular mechanisms, some studies through large-scale screening of fungal strains found that filamentous fungi through the secretion of organic acids, regulating acidic and alkaline phosphatase activity to neutralise soil pH to achieve the mobilisation of available phosphorus sources in the soil, in addition to increase the content of soluble phosphorus in the soil, some of the strains can also assist in the synthesis of phytohormones, such as indole acetic acid and abscisic acid, to promote the growth of the plant [83]. During arsenic stress, the endophytic mycorrhizal fungus *Serendipita indica* achieves the conversion of organic phosphorus to soluble phosphorus in the soil through rapid secretion of acid phosphatase, promotes the storage of polyphosphates, and reduces the toxicity of arsenic stress on the plant through competitive uptake with arsenic and pH changes [84,85]. The amount of available phosphorus was found to affect selenium uptake in winter wheat. When not inoculated with mycorrhizal fungi, an increase in the amount of phosphorus fertiliser would promote selenium uptake by the plant, and an overdose would inhibit this effect. After inoculation with mycorrhizal fungi of mycorrhizal fungi, selenium content in the root system and above-ground parts of winter wheat was higher than that of the uninoculated group regardless of the level of phosphorus fertiliser application, and the fungal mycelium network expanded the uptake range of the root system and improved the inter-root microenvironment to promote the plant’s growth [86] (Figure 4).

### 4.3. Biological Significance of Phosphate Metabolism of Pathogenic Fungi

Phosphate metabolism also plays an indispensable role in the pathogenicity of fungi. Under low-phosphorus conditions, pathogenic fungi can activate the expression of virulence factors by activating the phosphorus starvation response pathway. In *Candida albicans*, the transcription factor Pho4 is up-regulated under phosphorus-limited conditions, which induces the expression of proteases and other genes to enhance the invasive ability of the mycelium and its ability to damage the membrane of the host cell [6]; the deletion of myo-inositol pyrophosphate synthase VIP1 leads to disorders in energy metabolism, and the cells cannot sense the available phosphorus in a phosphorus starved state. available phosphorus in a phosphorus starvation state, the PHO signalling pathway remains active, lipid droplet accumulation, lipid peroxidation, decreased cell membrane integrity and permeability, and cell wall repair-related genes are up-regulated in the mutant strains, suggesting that the cells may be able to maintain their survival by remodelling the cell wall structure when the membrane structure is damaged [69].

In *Cryptococcus neoformans*, defective activation of the PHO pathway significantly reduces cellular energy metabolism and pathogenicity. Sustained activation of the PHO pathway leads to excessive phosphorus accumulation and elevated metal ion concentrations, enhanced sensitivity to metal ions, inhibition of calmodulin phosphatase signalling, which is partially restored by restricting phosphorus supply impaired polysaccharide pod synthesis, making the cells susceptible to clearance by host macrophages [8,52]. Polyphosphate plays a key role in this process, and when polyP synthesis and catabolism are blocked, the mutant strains have impaired in vitro adhesion, mycelium formation, and biofilm formation, and the strains were found to have drastically weakened pathogenicity, limited infection spread, and elevated host survival in animal models [6]. Excess phosphorus accumulation in rice leaves significantly increased the infestation rate of the rice blast fungus *Magnaporthe oryzae*, and high-phosphorus conditions not only favoured the formation of pathogenic fungal energy acquisition and infestation structures to provide nutrients for the pathogenic fungus, but also suppressed the host immune response, and the expression of pathogenic virulence factors rose [87]. In addition to the fungus itself affected by phosphorus concentration, some plant pathogenic fungi such as *Colletotrichum higginsianum* secreted effector proteins with Nudix hydrolase activity in the process of infestation and transported into plant cells, hydrolysed some of the phosphorus metabolism signalling molecules in the plant body to disrupt the phosphorus starvation system of the plant to enhance the colonisation and pathogenicity [88].

### 4.4. Fungal-Based Biostimulants Effectively Enhance Phosphorus Availability

In recent years, fungal-based biostimulants have attracted considerable attention for their ability to enhance phosphorus availability in soils. As previously discussed in this paper, typical phosphate-solubilising fungi (PSF), including *Aspergillus*, *Penicillium*, *Trichoderma*, and *Fusarium*, have the capacity to convert insoluble inorganic and organic phosphorus into plant-available forms by secreting organic acids (e.g., oxalic, citric, malic acids) and phosphatases. This process significantly enhances crop phosphorus uptake efficiency [71,89,90,91,92]. Beyond solubilising phosphorus to enhance its availability, these fungi indirectly boost plant productivity through mechanisms including promoting root development, regulating rhizosphere microbial community structure, and enhancing nutrient cycling. For instance, certain fungal biopesticides based on PSF have been shown to effectively increase available phosphorus and crop yields under both greenhouse and field conditions [71]. The combined application of PSF with arbuscular mycorrhizal fungi or organic amendments has been shown to exhibit pronounced synergistic effects [74], substantially enhancing soil microbial biomass, phosphatase activity, and long-term fertility [93]. It has been demonstrated that specific functional fungal communities (e.g., nematophagous and wood-decaying fungi) manifest dual ecological effects, namely the mobilisation of nutrients and the suppression of pathogens [72]. These can enhance phosphorus utilisation while inhibiting plant diseases, demonstrating potential for developing multifunctional fungal preparations. Nevertheless, the development of fungal biocontrol agents continues to be confronted by numerous challenges. These include the unstable environmental adaptability of strains, the lack of universal applicability in formulations, competition with indigenous microbial communities, and issues concerning the timeliness of application post-introduction. These issues warrant further attention.

## 5. Conclusions and Perspectives

Phosphates are not only essential nutrients for fungal growth but also participate in signal transduction and energy metabolism. The PHO pathway is subject to regulation by the Pho80–Pho85 CDK complex, which has been demonstrated to promote phosphate uptake and gene expression via Pho4 [6]. It is important to note that specific variations exist among fungi. C. glabrata exhibits a lower dependency on Pho4–Pho2, C. albicans possesses distinct N-terminal phosphorylation sites for Pho4, and C. neoformans can activate Pho4 at pH values above neutral. In the context of symbiotic fungi [8], bidirectional phosphotransporter proteins facilitate the exchange of phosphorus between the host organism and the fungus.

The SPX domain, present in Pho81, the VTC complex, and Xpr1, has been demonstrated to regulate PHO signalling and polyphosphate synthesis by binding inositol pyrophosphate. Polyphosphate functions as an intracellular phosphate reservoir, synthesised catalytically by the VTC complex and stored in vacuoles. It participates in phosphate buffering, stress responses, and virulence regulation [5,85,94]. As a result of the action of PPN/PPx phosphatases, the maintenance of homeostasis is ensured through the process of degradation, with the resultant regulation of metal ion balance and antioxidant capacity. In the case of C. neoformans, elevated phosphorus levels have been demonstrated to promote capsular formation, drug resistance, and cell wall synthesis [8]. Within symbiotic systems, transcription factors and transporters cooperate to regulate phosphorus exchange. Defective RiPT expression leads to polyphosphate overaccumulation and symbiotic structure degradation.

Despite the increasing insights into the PHO–SPX–polyP regulatory axis, its conservation and variation across fungal taxa remain unclear. Future research should focus on: The following three areas of research are of particular interest: (i) The molecular mechanisms of phosphorus sensing and signalling in non-model fungi; (ii) The coupled regulation of phosphorus metabolism with secondary metabolism, symbiotic relationships, and pathogenicity; (iii) The application of multi-omics approaches, advanced imaging techniques, and computational modelling to elucidate the dynamic regulation of phosphorus homeostasis. A comprehensive examination of these elements will facilitate enhanced comprehension of fungal ecological adaptation strategies and establish a theoretical framework for the advancement of biofertiliser and the sustainable utilisation of phosphorus within agricultural and natural ecosystems.

## 6. Materials and Methods

### 6.1. Literature Search and Selection

This review is based on a systematic search and synthesis of research articles published during the past five years (2020–2025). The primary databases used for literature retrieval included PubMed, Web of Science Core Collection, and Google Scholar. A systematic Boolean logic-based search strategy was applied to ensure comprehensive coverage of relevant studies. The main search keywords and their combinations were as follows: “fungal phosphate metabolism” OR “phosphate signaling” OR “PHO pathway” OR “SPX domain” OR “inositol pyrophosphate” OR “polyphosphate” OR “phosphate homeostasis” OR “phosphate transporter” OR “phosphate starvation.”

The search was restricted to peer-reviewed articles published in English. To further enhance the completeness and representativeness of the review, citation tracking was performed to identify additional key reviews and highly cited research papers relevant to the topic.

The inclusion criteria were as follows: (1) studies focused on fungi, including yeasts, filamentous fungi, pathogenic fungi, and symbiotic fungi; (2) studies investigating the molecular, genetic, or biochemical mechanisms of phosphate sensing, storage, signaling, or polyphosphate metabolism; and (3) publications from 2020 to 2025.

Exclusion criteria included non-fungal studies, non-peer-reviewed articles, conference abstracts, and opinion papers lacking experimental data.

### 6.2. Data Organization and Integration

All included studies were categorized and synthesized according to their major research themes, encompassing: (1) PHO pathway signaling and regulatory mechanisms; (2) SPX–PP-IP complex-mediated phosphate coupling and regulation; (3) mechanisms of polyphosphate synthesis and degradation; and (4) epigenetic regulation and its integration with phosphate metabolism.

Cross-validation among multiple studies was performed to ensure consistency and reliability of the summarized information. Key findings from independent reports were systematically compared and integrated to highlight consensus models, mechanistic advances, and conceptual developments in fungal phosphate metabolism and signaling.

## Data Availability

The original contributions presented in this study are included in the article. Further inquiries can be directed to the corresponding author.

## References

[1] Kritmetapak K., Kumar R. (2021). Phosphate as a Signaling Molecule. Calcif. Tissue Int..

[2] Bhadada S.K., Rao S.D. (2021). Role of Phosphate in Biomineralization. Calcif. Tissue Int..

[3] Puga M.I., Poza-Carrión C., Martinez-Hevia I., Perez-Liens L., Paz-Ares J. (2024). Recent Advances in Research on Phosphate Starvation Signaling in Plants. J. Plant Res..

[4] Dai S., Chen H., Shi Y., Xiao X., Xu L., Qin C., Zhu Y., Yi K., Lei M., Zeng H. (2024). PHOSPHATE1-Mediated Phosphate Translocation from Roots to Shoots Regulates Floral Transition in Plants. J. Exp. Bot..

[5] Ikeh M.A.C., Kastora S.L., Day A.M., Herrero-de-Dios C.M., Tarrant E., Waldron K.J., Banks A.P., Bain J.M., Lydall D., Veal E.A. (2016). Pho4 Mediates Phosphate Acquisition in *Candida albicans* and Is Vital for Stress Resistance and Metal Homeostasis. Mol. Biol. Cell.

[6] Ahmed Y., Ikeh M.A.C., MacCallum D.M., Day A.M., Waldron K., Quinn J. (2022). Blocking Polyphosphate Mobilization Inhibits Pho4 Activation and Virulence in the Pathogen *Candida albicans*. mBio.

[7] King W.R., Acosta-Zaldívar M., Qi W., Cherico N., Cooke L., Köhler J.R., Patton-Vogt J. (2023). Glycerophosphocholine Provision Rescues *Candida albicans* Growth and Signaling Phenotypes Associated with Phosphate Limitation. mSphere.

[8] Qu X., Bhalla K., Horianopoulos L.C., Hu G., Alcázar Magaña A., Foster L.J., Roque Da Silva L.B., Kretschmer M., Kronstad J.W. (2024). Phosphate Availability Conditions Caspofungin Tolerance, Capsule Attachment and Titan Cell Formation in *Cryptococcus neoformans*. Front. Fungal Biol..

[9] Suraby E.J., Agisha V.N., Dhandapani S., Sng Y.H., Lim S.H., Naqvi N.I., Sarojam R., Yin Z., Park B.S. (2023). Plant Growth Promotion under Phosphate Deficiency and Improved Phosphate Acquisition by New Fungal Strain, Penicillium Olsonii TLL1. Front. Microbiol..

[10] Xie X., Lai W., Che X., Wang S., Ren Y., Hu W., Chen H., Tang M. (2022). A SPX Domain-containing Phosphate Transporter from *Rhizophagus irregularis* Handles Phosphate Homeostasis at Symbiotic Interface of Arbuscular Mycorrhizas. New Phytol..

[11] Bhalla K., Qu X., Kretschmer M., Kronstad J.W. (2022). The Phosphate Language of Fungi. Trends Microbiol..

[12] Li H., Smith S.E., Holloway R.E., Zhu Y., Smith F.A. (2006). Arbuscular Mycorrhizal Fungi Contribute to Phosphorus Uptake by Wheat Grown in a Phosphorus-fixing Soil Even in the Absence of Positive Growth Responses. New Phytol..

[13] Wang G., Jin Z., George T.S., Feng G., Zhang L. (2023). Arbuscular Mycorrhizal Fungi Enhance Plant Phosphorus Uptake through Stimulating Hyphosphere Soil Microbiome Functional Profiles for Phosphorus Turnover. New Phytol..

[14] Van’T Padje A., Werner G.D.A., Kiers E.T. (2021). Mycorrhizal Fungi Control Phosphorus Value in Trade Symbiosis with Host Roots When Exposed to Abrupt ‘Crashes’ and ‘Booms’ of Resource Availability. New Phytol..

[15] Köhler J., Yang N., Pena R., Raghavan V., Polle A., Meier I.C. (2018). Ectomycorrhizal Fungal Diversity Increases Phosphorus Uptake Efficiency of European Beech. New Phytol..

[16] Ji B., Bever J.D. (2016). Plant Preferential Allocation and Fungal Reward Decline with Soil Phosphorus: Implications for Mycorrhizal Mutualism. Ecosphere.

[17] Yang G., Liu N., Lu W., Wang S., Kan H., Zhang Y., Xu L., Chen Y. (2014). The Interaction between Arbuscular Mycorrhizal Fungi and Soil Phosphorus Availability Influences Plant Community Productivity and Ecosystem Stability. J. Ecol..

[18] Chen E., Liao H., Chen B., Peng S. (2020). Arbuscular Mycorrhizal Fungi Are a Double-edged Sword in Plant Invasion Controlled by Phosphorus Concentration. New Phytol..

[19] Pánek M., Vlková T., Michalová T., Borovička J., Tedersoo L., Adamczyk B., Baldrian P., Lopéz-Mondéjar R. (2024). Variation of Carbon, Nitrogen and Phosphorus Content in Fungi Reflects Their Ecology and Phylogeny. Front. Microbiol..

[20] Takado M., Komamura T., Nishimura T., Ohkubo I., Ohuchi K., Matsumoto T., Takeda K. (2023). Phosphate Uptake Restriction, Phosphate Export, and Polyphosphate Synthesis Contribute Synergistically to Cellular Proliferation and Survival. J. Biol. Chem..

[21] Groth B., Lee Y.-C., Huang C.-C., McDaniel M., Huang K., Lee L.-H., Lin S.-J. (2023). The Histone Deacetylases Hst1 and Rpd3 Integrate De Novo NAD+ Metabolism with Phosphate Sensing in *Saccharomyces cerevisiae*. Int. J. Mol. Sci..

[22] Chen Y., Farooq A., Wei X., Qin L., Wang Y., Zhang L., Xiang Q., Zhao K., Yu X., Chen Q. (2025). Transcriptomic and Metabolomic Analysis of Recalcitrant Phosphorus Solubilization Mechanisms in *Trametes gibbosa*. Front. Microbiol..

[23] Ming Yip H., Cheng S., Olson E.J., Crone M., Maerkl S.J. (2023). Perfect Adaptation Achieved by Transport Limitations Governs the Inorganic Phosphate Response in *S. Cerevisiae*. Proc. Natl. Acad. Sci. USA.

[24] Austin S., Mayer A. (2020). Phosphate Homeostasis—A Vital Metabolic Equilibrium Maintained Through the INPHORS Signaling Pathway. Front. Microbiol..

[25] Pipercevic J., Kohl B., Gerasimaite R., Comte-Miserez V., Hostachy S., Müntener T., Agustoni E., Jessen H.J., Fiedler D., Mayer A. (2023). Inositol Pyrophosphates Activate the Vacuolar Transport Chaperone Complex in Yeast by Disrupting a Homotypic SPX Domain Interaction. Nat. Commun..

[26] Huang Z., Zhang S., Chen R., Zhu Q., Shi P., Shen Y. (2023). The Transporter PHO84/NtPT1 Is a Target of Aluminum to Affect Phosphorus Absorption in *Saccharomyces cerevisiae* and *Nicotiana tabacum* L.. Metallomics.

[27] Henry T.C., Power J.E., Kerwin C.L., Mohammed A., Weissman J.S., Cameron D.M., Wykoff D.D. (2011). Systematic Screen of *Schizosaccharomyces pombe* Deletion Collection Uncovers Parallel Evolution of the Phosphate Signal Transduction Pathway in Yeasts. Eukaryot. Cell.

[28] Snyder L.F., O’Brien E.M., Zhao J., Liang J., Bruce B.J., Zhang Y., Zhu W., Cassier T., Schnicker N.J., Zhou X. (2025). Divergence in a Eukaryotic Transcription Factor’s Co-TF Dependence Involves Multiple Intrinsically Disordered Regions 2024. Nat. Commun..

[29] Komamura T., Nishimura T., Ohta N., Takado M., Matsumoto T., Takeda K. (2025). The Putative Polyamine Transporter Shp2 Facilitates Phosphate Export in an Xpr1-Independent Manner and Contributes to High Phosphate Tolerance. J. Biol. Chem..

[30] Speedman D., Sauer D.B. (2024). Pho Pictures Provide Powerful Perspectives of Phosphate Importing Proteins. Structure.

[31] Choi J., Rajagopal A., Xu Y.-F., Rabinowitz J.D., O’Shea E.K. (2017). A Systematic Genetic Screen for Genes Involved in Sensing Inorganic Phosphate Availability in *Saccharomyces cerevisiae*. PLoS ONE.

[32] Ogawa N., DeRisi J., Brown P.O. (2000). New Components of a System for Phosphate Accumulation and Polyphosphate Metabolism in *Saccharomyces cerevisiae* Revealed by Genomic Expression Analysis. Mol. Biol. Cell.

[33] Secco D., Wang C., Shou H., Whelan J. (2012). Phosphate Homeostasis in the Yeast *Saccharomyces cerevisiae*, the Key Role of the SPX Domain-containing Proteins. FEBS Lett..

[34] Carter-O’Connell I., Peel M.T., Wykoff D.D., O’Shea E.K. (2012). Genome-Wide Characterization of the Phosphate Starvation Response in *Schizosaccharomyces pombe*. BMC Genom..

[35] Estill M., Kerwin-Iosue C.L., Wykoff D.D. (2015). Dissection of the PHO Pathway in *Schizosaccharomyces pombe* Using Epistasis and the Alternate Repressor Adenine. Curr. Genet..

[36] Chabert V., Kim G.-D., Qiu D., Liu G., Michaillat Mayer L., Jamsheer K M., Jessen H.J., Mayer A. (2023). Inositol Pyrophosphate Dynamics Reveals Control of the Yeast Phosphate Starvation Program through 1,5-IP8 and the SPX Domain of Pho81. eLife.

[37] Ghosh S., Sanchez A.M., Schwer B., Prucker I., Jork N., Jessen H.J., Shuman S. (2024). Activities and Genetic Interactions of Fission Yeast Aps1, a Nudix-Type Inositol Pyrophosphatase and Inorganic Polyphosphatase. mBio.

[38] Bednor L., Sanchez A.M., Garg A., Shuman S., Schwer B. (2024). Genetic Suppressor Screen Identifies Tgp1 (Glycerophosphocholine Transporter), Kcs1 (IP_6_ Kinase), and Plc1 (Phospholipase C) as Determinants of Inositol Pyrophosphate Toxicosis in Fission Yeast. mBio.

[39] Pascual-Ortiz M., Walla E., Fleig U., Saiardi A. (2021). The PPIP5K Family Member Asp1 Controls Inorganic Polyphosphate Metabolism in S. Pombe. J. Fungi.

[40] Schwer B., Prucker I., Sanchez A.M., Babor J., Jessen H.J., Shuman S. (2025). Tandem Inactivation of Inositol Pyrophosphatases Asp1, Siw14, and Aps1 Illuminates Functional Redundancies in Inositol Pyrophosphate Catabolism in Fission Yeast. mBio.

[41] Sunder S., Bauman J.S., Decker S.J., Lifton A.R., Kumar A. (2024). The Yeast AMP-Activated Protein Kinase Snf1 Phosphorylates the Inositol Polyphosphate Kinase Kcs1. J. Biol. Chem..

[42] Gogianu L.I., Ruta L.L., Farcasanu I.C. (2024). Kcs1 and Vip1: The Key Enzymes behind Inositol Pyrophosphate Signaling in *Saccharomyces cerevisiae*. Biomolecules.

[43] Guan Z., Chen J., Liu R., Chen Y., Xing Q., Du Z., Cheng M., Hu J., Zhang W., Mei W. (2023). The Cytoplasmic Synthesis and Coupled Membrane Translocation of Eukaryotic Polyphosphate by Signal-Activated VTC Complex. Nat. Commun..

[44] Martín J.F. (2023). Interaction of Calcium Responsive Proteins and Transcriptional Factors with the PHO Regulon in Yeasts and Fungi. Front. Cell Dev. Biol..

[45] Schwer B., Garg A., Sanchez A.M., Bernstein M.A., Benjamin B., Shuman S. (2022). Cleavage-Polyadenylation Factor Cft1 and SPX Domain Proteins Are Agents of Inositol Pyrophosphate Toxicosis in Fission Yeast. mBio.

[46] Benjamin B., Garg A., Jork N., Jessen H.J., Schwer B., Shuman S. (2022). Activities and Structure-Function Analysis of Fission Yeast Inositol Pyrophosphate (IPP) Kinase-Pyrophosphatase Asp1 and Its Impact on Regulation of *Pho1* Gene Expression. mBio.

[47] Schwer B., Innokentev A., Sanchez A.M., Garg A., Shuman S. (2024). Suppression of Inositol Pyrophosphate Toxicosis and Hyper-Repression of the Fission Yeast *PHO* Regulon by Loss-of-Function Mutations in Chromatin Remodelers Snf22 and Sol1. mBio.

[48] Lieleg C., Novacic A., Musladin S., Schmid A., Akpinar G.G., Barbaric S., Korber P. (2023). Nucleosome Remodeling at the Yeast PHO8 and PHO84 Promoters without the Putatively Essential SWI/SNF Remodeler. Int. J. Mol. Sci..

[49] Garg A., Sanchez A.M., Schwer B., Shuman S. (2024). Factors Governing the Transcriptome Changes and Chronological Lifespan of Fission Yeast during Phosphate Starvation. J. Biol. Chem..

[50] Camblong J., Iglesias N., Fickentscher C., Dieppois G., Stutz F. (2007). Antisense RNA Stabilization Induces Transcriptional Gene Silencing via Histone Deacetylation in S. Cerevisiae. Cell.

[51] Chaves-Arquero B., Pérez-Cañadillas J.M. (2023). The Nrd1–Nab3–Sen1 Transcription Termination Complex from a Structural Perspective. Biochem. Soc. Trans..

[52] Bowring B.G., Sethiya P., Desmarini D., Lev S., Tran Le L., Bahn Y.-S., Lee S.-H., Toh-e A., Proschogo N., Savage T. (2023). Dysregulating PHO Signaling via the CDK Machinery Differentially Impacts Energy Metabolism, Calcineurin Signaling, and Virulence in *Cryptococcus neoformans*. mBio.

[53] Xing Y., Xu K., Li S., Cao L., Nan Y., Li Q., Li W., Hong Z. (2021). A Single-Domain Antibody-Based Anti-PSMA Recombinant Immunotoxin Exhibits Specificity and Efficacy for Prostate Cancer Therapy. Int. J. Mol. Sci..

[54] Liu W., Wang J., Comte-Miserez V., Zhang M., Yu X., Chen Q., Jessen H.J., Mayer A., Wu S., Ye S. (2023). Cryo-EM Structure of the Polyphosphate Polymerase VTC Reveals Coupling of Polymer Synthesis to Membrane Transit. EMBO J..

[55] Tomashevsky A., Kulakovskaya E., Trilisenko L., Kulakovskiy I.V., Kulakovskaya T., Fedorov A., Eldarov M. (2021). VTC4 Polyphosphate Polymerase Knockout Increases Stress Resistance of *Saccharomyces cerevisiae* Cells. Biology.

[56] Gerasimaitė R., Mayer A. (2017). Ppn2, a Novel Zn2+-Dependent Polyphosphatase in the Acidocalcisome-like Yeast Vacuole. J. Cell Sci..

[57] Eliseeva I.A., Ryazanova L., Ledova L., Zvonarev A., Valiakhmetov A., Suntsova M., Modestov A., Buzdin A., Lyabin D.N., Kulakovskiy I.V. (2024). Ppn2 Polyphosphatase Improves the Ability of S. Cerevisiae to Grow in Mild Alkaline Medium. J. Fungi.

[58] Andreeva N., Ryazanova L., Ledova L., Trilisenko L., Kulakovskaya T. (2022). Stress Resistance of *Saccharomyces cerevisiae* Strains Overexpressing Yeast Polyphosphatases. Stresses.

[59] Hürlimann H.C., Stadler-Waibel M., Werner T.P., Freimoser F.M. (2007). Pho91 Is a Vacuolar Phosphate Transporter That Regulates Phosphate and Polyphosphate Metabolism in *Saccharomyces cerevisiae*. Mol. Biol. Cell.

[60] Schneider S., Kühlbrandt W., Yildiz Ö. (2024). Complementary Structures of the Yeast Phosphate Transporter Pho90 Provide Insights into Its Transport Mechanism. Structure.

[61] Farofonova V., Andreeva N., Kulakovskaya E., Karginov A., Agaphonov M., Kulakovskaya T. (2023). Multiple Effects of the PHO91 Gene Knockout in Ogataea Parapolymorpha. Folia Microbiol..

[62] He Q., Zhang R., Tury S., Courgnaud V., Liu F., Battini J., Li B., Chen Q. (2025). Structural Basis of Phosphate Export by Human XPR1. Nat. Commun..

[63] Kulakovskaya E., Zvonarev A., Kulakovskaya T. (2023). PHM6 and PHM7 Genes Are Essential for Phosphate Surplus in the Cells of *Saccharomyces cerevisiae*. Arch. Microbiol..

[64] Kim G.-D., Qiu D., Jessen H.J., Mayer A. (2023). Metabolic Consequences of Polyphosphate Synthesis and Imminent Phosphate Limitation. mBio.

[65] Schoeppe R., Waldmann M., Jessen H.J., Renné T. (2024). An Update on Polyphosphate In Vivo Activities. Biomolecules.

[66] Popova Y., Thayumanavan P., Lonati E., Agrochão M., Thevelein J.M. (2010). Transport and Signaling through the Phosphate-Binding Site of the Yeast Pho84 Phosphate Transceptor. Proc. Natl. Acad. Sci. USA.

[67] Couso I., Pérez-Pérez M.E., Ford M.M., Martínez-Force E., Hicks L.M., Umen J.G., Crespo J.L. (2020). Phosphorus Availability Regulates TORC1 Signaling via LST8 in Chlamydomonas. Plant Cell.

[68] Park Y., Malliakas C.D., Zhou Q., Gu A.Z., Aristilde L. (2021). Molecular Coordination, Structure, and Stability of Metal-Polyphosphate Complexes Resolved by Molecular Modeling and X-Ray Scattering: Structural Insights on the Biological Fate of Polyphosphate. Environ. Sci. Technol..

[69] Peng X., Ma C., Feng Y., Zhang B., Zhu M., Ma T., Yu Q., Li M. (2022). Phosphate Starvation by Energy Metabolism Disturbance in Candida albicansvip1Δ/Δ Induces Lipid Droplet Accumulation and Cell Membrane Damage. Molecules.

[70] Pang F., Li Q., Solanki M.K., Wang Z., Xing Y.-X., Dong D.-F. (2024). Soil Phosphorus Transformation and Plant Uptake Driven by Phosphate-Solubilizing Microorganisms. Front. Microbiol..

[71] Ma Y., Chen S., Liu S., Guo L., Zhang C., Ye X., Tian D. (2025). Phosphate Solubilizing Fungi Enhance Insoluble Phosphate Dissolution via Organic Acid Production: Mechanisms and Applications. Front. Microbiol..

[72] Vera-Morales M., López Medina S.E., Naranjo-Morán J., Quevedo A., Ratti M.F. (2023). Nematophagous Fungi: A Review of Their Phosphorus Solubilization Potential. Microorganisms.

[73] Gadd G.M. (2021). Fungal Biomineralization. Curr. Biol..

[74] Watkinson S., Bebber D., Darrah P., Fricker M., Tlalka M., Boddy L., Gadd G.M. (2006). The Role of Wood Decay Fungi in the Carbon and Nitrogen Dynamics of the Forest Floor. Fungi in Biogeochemical Cycles.

[75] Tian D., Chen H., De Oliveira Mendes G., Feng Y. (2023). Editorial: Biotechnology of Phosphate Solubilizing Microorganisms for Metabolites Regulation: Present and Future. Front. Bioeng. Biotechnol..

[76] Arias R.M., Heredia Abarca G., Del Carmen Perea Rojas Y., De La Cruz Elizondo Y., García Guzman K.Y. (2023). Selection and Characterization of Phosphate-Solubilizing Fungi and Their Effects on Coffee Plantations. Plants.

[77] Sbrana C., Agnolucci M., Avio L., Giovannini L., Palla M., Turrini A., Giovannetti M. (2022). Mycorrhizal Symbionts and Associated Bacteria: Potent Allies to Improve Plant Phosphorus Availability and Food Security. Front. Microbiol..

[78] Shi J., Zhao B., Zheng S., Zhang X., Wang X., Dong W., Xie Q., Wang G., Xiao Y., Chen F. (2021). A Phosphate Starvation Response-Centered Network Regulates Mycorrhizal Symbiosis. Cell.

[79] Das D., Gutjahr C. (2022). Old Dog, New Trick: The PHR-SPX System Regulates Arbuscular Mycorrhizal Symbiosis. Mol. Plant.

[80] Srivastava R., Roychowdhury A., Kumar R. (2022). Host SPX-PHR Regulatory Circuit: The Molecular Dynamo Steering Mycorrhization in Plants. Plant Cell Rep..

[81] Zhang S., Nie Y., Fan X., Wei W., Chen H., Xie X., Tang M. (2023). A Transcriptional Activator from *Rhizophagus irregularis* Regulates Phosphate Uptake and Homeostasis in AM Symbiosis during Phosphorous Starvation. Front. Microbiol..

[82] Singh N.R.R., Roychowdhury A., Srivastava R., Akash, Gaganan G.A., Parida A.P., Kumar R. (2023). Silencing of SlSPX1 and SlSPX2 Promote Growth and Root Mycorrhization in Tomato (*Solanum lycopersicum* L.) Seedlings. Plant Sci..

[83] Brazhnikova Y.V., Shaposhnikov A.I., Sazanova A.L., Belimov A.A., Mukasheva T.D., Ignatova L.V. (2022). Phosphate Mobilization by Culturable Fungi and Their Capacity to Increase Soil P Availability and Promote Barley Growth. Curr. Microbiol..

[84] Kushwaha A.S., Ahmad I., Lata S., Padalia K., Yadav A.K., Kumar M. (2024). Mycorrhizal Fungus Serendipita Indica-Associated Acid Phosphatase Rescues the Phosphate Nutrition with Reduced Arsenic Uptake in the Host Plant under Arsenic Stress. Ecotoxicol. Environ. Saf..

[85] Kushwaha A.S., Thakur R.S., Patel D.K., Kumar M. (2022). Impact of Arsenic on Phosphate Solubilization, Acquisition and Poly-Phosphate Accumulation in Endophytic Fungus Serendipita Indica. Microbiol. Res..

[86] Li J., Liu R., Zhang C., Yang J., Lyu L., Shi Z., Man Y.B., Wu F. (2022). Selenium Uptake and Accumulation in Winter Wheat as Affected by Level of Phosphate Application and Arbuscular Mycorrhizal Fungi. J. Hazard. Mater..

[87] Martín-Cardoso H., Bundó M., Val-Torregrosa B., San Segundo B. (2024). Phosphate Accumulation in Rice Leaves Promotes Fungal Pathogenicity and Represses Host Immune Responses during Pathogen Infection. Front. Plant Sci..

[88] McCombe C.L., Wegner A., Wirtz L., Zamora C.S., Casanova F., Aditya S., Greenwood J.R., De Paula S., England E., Shang S. (2025). Plant Pathogenic Fungi Hijack Phosphate Signaling with Conserved Enzymatic Effectors. Science.

[89] Li Z., Bai T., Dai L., Wang F., Tao J., Meng S., Hu Y., Wang S., Hu S. (2016). A Study of Organic Acid Production in Contrasts between Two Phosphate Solubilizing Fungi: Penicillium Oxalicum and Aspergillus Niger. Sci. Rep..

[90] Ferreyra-Suarez D., García-Depraect O., Castro-Muñoz R. (2024). A Review on Fungal-Based Biopesticides and Biofertilizers Production. Ecotoxicol. Environ. Saf..

[91] García-Berumen J.A., Flores De La Torre J.A., De Los Santos-Villalobos S., Espinoza-Canales A., Echavarría-Cháirez F.G., Gutiérrez-Bañuelos H. (2025). Phosphorus Dynamics and Sustainable Agriculture: The Role of Microbial Solubilization and Innovations in Nutrient Management. Curr. Res. Microb. Sci..

[92] Wang C., Pan G., Lu X., Qi W. (2023). Phosphorus Solubilizing Microorganisms: Potential Promoters of Agricultural and Environmental Engineering. Front. Bioeng. Biotechnol..

[93] Eichler-Löbermann B., Blossei J., Kim D.-G. (2025). Microbial Strategies to Alleviate Phosphorus Deficiency in African Smallholder Farms: Inoculation and Soil Microbiome Enhancement. Plant Soil.

[94] Köhler J.R., Acosta-Zaldívar M., Qi W. (2020). Phosphate in Virulence of *Candida albicans* and *Candida glabrata*. J. Fungi.

